# Composition and origins of decorated glass from Umayyad Cordoba (Spain)

**DOI:** 10.1186/s40494-021-00505-4

**Published:** 2021-03-12

**Authors:** Jorge De Juan Ares, Yasmina Cáceres Gutiérrez, Maudilio Moreno Almenara, Nadine Schibille

**Affiliations:** 1 IRAMAT-CEB, UMR5060, CNRS/Université d’Orléans, 3D, Rue de la Férollerie, 45071 Orléans, France; 2 Departamento de Prehistoria, Historia Antigua y Arqueología, Universidad Complutense de Madrid, C/ Profesor Aranguren, s/n. Ciudad Universitaria, 28040 Madrid, Spain; 3 Independent researcher, Córdoba, Spain

**Keywords:** LA-ICP-MS, Islamic glass, Soda-ash glass, Soda-ash lead glass, Al-Andalus, Mould-blowing, Relief-cutting

## Abstract

Recent archaeological excavations carried out in the western suburbs of Cordoba (Spain) brought to light numerous fragments of archaeological glass from the caliphal period (929–1031 CE). The typological and compositional analysis by laser ablation inductively coupled plasma mass spectrometry (LA-ICP-MS) of 66 fragments enabled the identification of different types of base glass and glass working techniques, identifying local productions, imports and decorative imitations of eastern models. The studied fragments include Mesopotamian, Levantine, Egyptian and possibly Sicilian soda-rich plant ash glass categories, and various glass-decorating techniques such as mould-blowing, pressing, cutting, staining or gilding. The systematic comparison of the trace element patterns of several relief-cut objects identified both imported ware and a locally manufactured sample, whereas all mould-blown pieces were made from locally sourced raw materials. Iberian glassworkers seem to have preferred mould-blowing, probably because of the distinct working properties of locally available high lead glass. The results thus confirm the continuous long-distance exchange of vitreous material, as well as the existence of multiple glassmaking centres in the Iberian Peninsula, illustrating a link between secondary glass working techniques and chemical composition. Furthermore, the identification of several fragments belonging to the same object based on chemical composition allowed us to reconstruct entire vessels and thereby expand the repertoire of known typologies circulating in Umayyad Spain.

**Supplementary Information:**

The online version contains supplementary material available at 10.1186/s40494-021-00505-4.

## Introduction

Over the last 5 years, there has been significant progress in the study of glass compositions in the medieval Iberian Peninsula. While Roman and late antique natron-type glass from Egypt and the Levant continued to prevail in the sixth and seventh centuries, glassmaking experienced a significant increase in recycling at the end of the Visigothic period, probably due to insufficient fresh glass supplies [1]. Glass finds of the early Islamic period are relatively scarce in al-Andalus, and recycling of natron glass seems to have been the main source of supply also during the first century of Islamic rule [2, 3]. Recent work on the elemental and isotopic composition of early Umayyad glass assemblages from the late eighth and early ninth centuries identified a novel glassmaking recipe in Cordoba, using lead slag as the main ingredient [3]. This new glassmaking technology appeared independently of contemporary trends in the Islamic east, but instead bears some resemblance to high lead glass from Carolingian Europe [3]. Different types of lead glass spread throughout Europe and the Islamic world at more or less the same time, but to what extent these lead glassmaking practices were connected is difficult to tell at present [4–6]. In addition to recycling and the local production of lead glass, there is still evidence of glass imports in al-Andalus in the form of early Islamic natron type Egypt 2 as well as soda-rich plant ash glass of Levantine and Mesopotamian origins. These imports, however, pertain to finished objects rather than consignments of raw glass.

With the arrival of Islamic rule in al-Andalus, the repertoire of vessel types and glass decorations gradually changed, presumably inspired by glassmaking in the Islamic East [7]. In al-Andalus, the same range of glass decorating techniques can be found as in the rest of the Islamic Mediterranean, even though the absolute number of finds tends to be more limited. By far the most common forming technique in the Umayyad Caliphate of Cordoba (929–1031 CE) was mould-blowing. Less frequent are relief-cut decorations, which are usually associated with the richest archaeological contexts. Much less common are other decorative techniques such as imprints, staining, gilding and coloured threads [7–9]. These techniques all have parallels in the Islamic East and have been interpreted either as imports or as local productions that replicate oriental models. It has been suggested that mould-blown decorations may have been locally produced, given their abundance in al-Andalus [10]. On the other hand, the majority of relief-cut decorated vessels are believed to be imports from Egypt, Syria, and/or Mesopotamia, with the possible exception of a few individual objects that differ in execution and style [9–14].

To determine the origin of the raw materials used for the production of the decorated glasses from Umayyad Cordoba and by extension the location of their production, we can draw on compositional analyses. For example, recent analytical data suggest that a stained-glass fragment found in Šaqunda (Cordoba) from the late eighth or early ninth century was imported from Egypt [15]. Thanks to recent advances in the compositional classification of early Islamic glass assemblages and the expanding corpus of analytical data [16–25], regional variations have been identified that allow us to trace the scale of the trade of glass and the extent of the economic, cultural and technological exchange between the eastern Islamic world and Umayyad al-Andalus. To investigate whether the decorated glass fragments recovered from tenth-century Cordoba are of a local production or imports, we have thus quantitatively analysed the chemical composition of 66 glass samples from domestic contexts. Combining detailed typological assessments with compositional analyses reveals a possible link between decorative techniques, composition and provenance. This in turn may help identify technical reasons underlying the choice of materials in connection with forming techniques, as well as mechanisms driving the transfer of technologies and glass working skills.

### Archaeological context

**Fig. 1:**
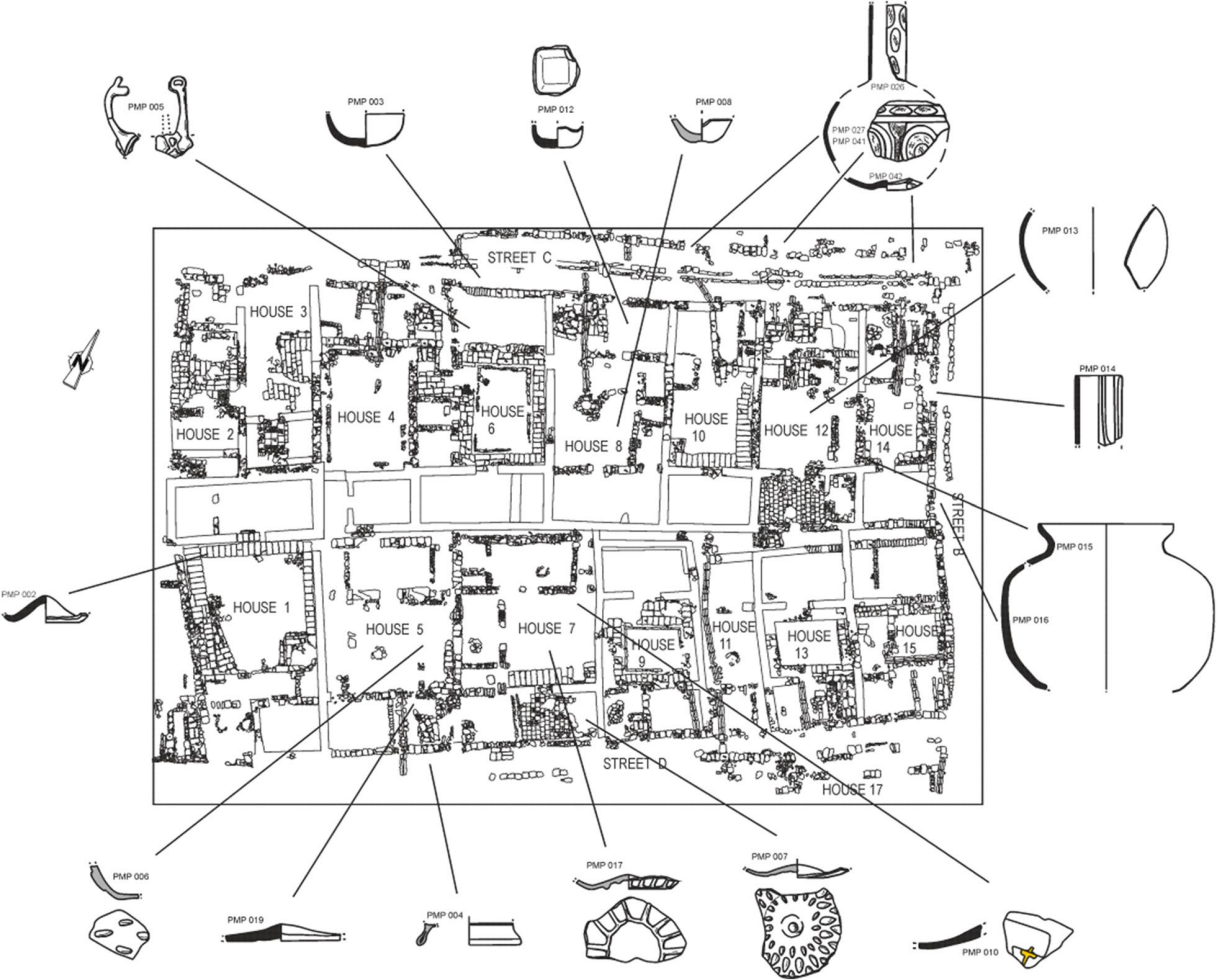
Excavated area with reference to the location of the find spots of some glass finds

In 2005, a rescue excavation of 2000 m^2^ was conducted in the so-called *Piscinas Municipales de Poniente* (PMP) in the *Arrabales Occidentales*, the western suburbs of the medieval Islamic city of Cordoba. During the caliphal period, this area underwent an urban development in a previously unoccupied zone. The archaeological research revealed a group of twenty-eight exceptionally well-preserved houses, fifteen of which were excavated in full (Fig. 1). A wide range of associated archaeological finds such as ceramics, metals and coins were recovered from these domestic contexts with high stratigraphic reliability. The lifetime of these dwellings was short. They were built around the middle of the tenth century and destroyed in the first third of the eleventh century during the conflicts that ravaged the city of Cordoba at the end of the caliphate. A dirham minted in the name of Hixam II (393-399H/1015-1021 CE), without date, combined with historical and archaeological data, gives a time of abandonment between the years 1015 and 1031 CE. After the end of the caliphate, the area was turned into farmland which it remained until the time of the archaeological excavations [26, 27]. Most of the vitreous material was found inside the houses and the surrounding streets linked to the destruction layers, few came from later natural filling levels with hardly any intrusions from subsequent periods (Fig. 1, Additional file 1: Table S1).

### Materials

**Fig. 2:**
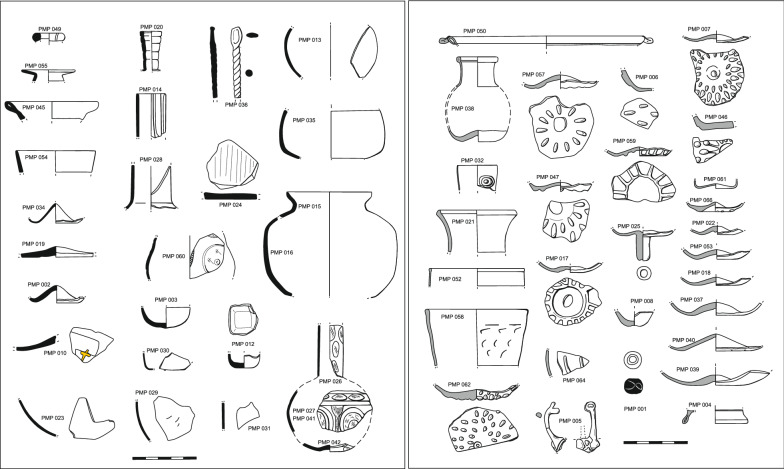
Glass finds separated by composition, showing plant ash glasses (left) and soda-ash lead glasses (right)

66 glass samples were taken from each of the discrete groups of fragments that were recovered together and stored in separate bags and/or boxes at the Gerencia de Urbanismo del Ayuntamiento de Córdoba (GMU), or that appeared to belong to different objects. This sampling strategy did not prevent the analysis of a few samples that turned out to belong to the same object. This accidental duplication had the additional effect that the complete shape of some vessels could be reconstructed (Fig. 2). Among the objects are bottles, unguentaria, bowls, beakers, lamps, flat glass, a small glass spoon and one bead. The glass is mostly colourless with some greenish, bluish or yellowish tinges, one blue, one purple and one black opaque fragment (Additional file 1: Table S1). Despite the relatively small number of vessels, the main glass working processes known from the Islamic world for shaping and decorating glass are represented in Cordoba, including mould-blowing, cutting, pressing, the application of white trails and gilding. As usual with other contemporary glass assemblages from al-Andalus, the most common technique is mould-blowing and the decorations include drops or radial ribs, mainly on cups or bowls. The few relief-cut decorations show mainly circular and linear motifs. Among the finds are only two fragments with embossed motifs (circles and slanting lines), one fragment with gilded remains and one glass bead with white trails (Fig. 2).

### Methods

Small fragments of the 66 samples were mounted in epoxy resin, polished and analysed by laser ablation inductively coupled mass spectrometry (LA-ICP-MS) at IRAMAT-CEB in Orléans following the protocols for glass analysis established in the laboratory [28]. Laser ablation was performed with a Resonetic UV laser microprobe (193 Excimer laser coupled with a Thermofisher Element XR mass spectrometer) operating at 6 mJ with 10 Hz frequency and a spot size of 80 μm to 100 μm for plant ash glass depending on manganese concentrations and 60 μm for high lead glass to avoid saturation. A pre-ablation of 15 s was followed by 27 s sample time. An argon/helium gas flow at a rate of 1 l/min Ar and 1 l/Ar + 0.65 l/min He transports the ablated material to the plasma torch where it is dissociated and ionised at a temperature of 8000 °C. The calculation of quantitative results was based on internal and international standards (NIST SRM610, Corning B, C and D). Detection limits range from 0.1% to 0.01% and from 20 to 500 ppb for major and trace elements, respectively. To establish accuracy and precision of the analyses, reference glasses (NIST SRM612, Corning A, B, D) were repeatedly analysed along with the archaeological samples (Additional file 1: Table S2).

## Results

**Fig. 3:**
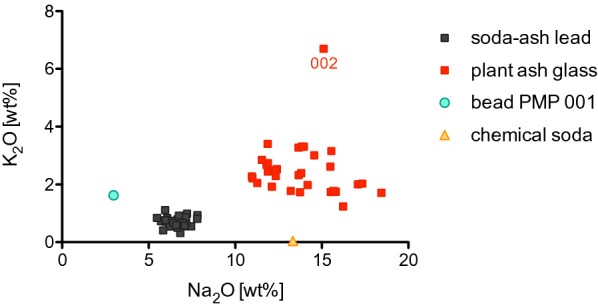
K_2_O versus Na_2_O concentrations of the glass from *Piscinas Municipales de Poniente* (PMP). Differences in the alkali concentrations identify the two main groups and single out a lead silica bead and a modern sample produced with chemical soda

The compositional results show that the assemblage consists of two major compositional groups that differ in terms of their alkali contents (Fig. 3): soda-ash lead glass (PMP Pb; n = 32) and different types of plant-ash glass (PMP-L, PMP-M, PMP-I, PMP-S; n = 32), in addition to a single high lead bead with a black body that was analysed, and a fragment of modern glass not further discussed here (Additional file 1: Table S1).

The 66 samples analysed seem to represent only 60 different archaeological objects. The analyses show that three groups of samples (PMP 15/16; PMP 023/029/044; PMP 026/027/041/042) came from three objects as their chemical compositions are within the margins of the instrumental precision and can thus be considered identical [29]. This has made it possible to reconstruct the complete shape of two vessels (Fig. 2: PMP 026/027/041/042 & PMP 15/16), and the identification of three body fragments of the same globular vessel (PMP 023/029/044).

### Soda-ash lead glass

The soda-ash lead glasses from Piscinas Municipales have a very uniform composition (Table 1). This glass consists mainly of lead (PbO ≈ 44%), silica (SiO_2_ ≈ 41%) and soda (Na_2_O ≈ 6.6%), with some lime (CaO ≈ 2%), magnesia (MgO ≈ 1%) and alumina (Al_2_O_3_ ≈ 1%). All other elements remain below 1%, except for chlorine that is exceptionally high, ranging from about 1.3% to 2.2%. Further features include elevated arsenic, silver, tin, antimony and bismuth contents (Additional file 1: Table S1) that suggest the use of litharge from silver cupellation as the main lead-bearing raw material [3, 30, 31]. Most of the soda-ash lead glass samples are colourless, while some show a greenish or yellowish tinge. They all display a characteristic corrosion of an opaque, white weathered surface over a silvery layer that makes them easily identifiable. A third of the soda-ash lead glasses has moulded decorations of different shapes, mostly drops but also lines and ribs. One sample (PMP 002) has impressed concentric circles, and another fragment (PMP 058) is decorated with slanting lines (Fig. 2). The remaining samples are undecorated, but pontil marks clearly identifiable in some of the bases leave no doubt that they were mould-blown [32].

**Table 1:** Average composition and standard deviation (σ) of the soda-ash lead and plant ash glass groups from Piscinas Municipales de Poniente

		Deco	Wt%												ppm						
			Na_2_O	MgO	Al_2_O_3_	SiO_2_	P_2_O_5_	Cl	K_2_O	CaO	TiO_2_	MnO	Fe_2_O_3_	PbO	Li	B	Cr	Sr	Zr	La	Th
PMP Pb soda-ash lead	mean (n = 32)	Mould blown & impressed	6.60	1.12	1.04	40.8	0.37	1.78	0.71	2.01	0.05	0.63	0.55	43.9	14.9	59.8	5.36	79.2	15.3	2.95	0.74
	σ		0.61	0.21	0.24	1.5	0.07	0.20	0.17	0.32	0.01	0.34	0.13	1.9	5.2	8.8	1.30	23.3	4.3	0.73	0.18
PMP-L Levantine	mean (n = 14)	Cut decorations	12.4	3.13	1.75	68.3	0.26	0.76	2.41	9.45	0.08	0.91	0.44	0.00	6.36	85.8	10.1	524	32.9	5.84	0.77
	σ		1.1	0.36	0.19	1.5	0.04	0.07	0.31	1.11	0.01	0.25	0.08	0.01	0.68	8.7	2.1	92	3.3	0.44	0.08
PMP-M Mesopotamian	PMP 010	Gold leaf	12.3	5.17	1.00	71.0	0.07	0.63	2.49	6.46	0.03	0.43	0.26	0.00	22.8	57.4	10.8	386	23.2	2.89	0.77
	PMP 054		15.5	4.93	2.58	66.1	0.14	0.64	3.16	5.44	0.06	0.83	0.44	0.00	16.4	117	23.0	455	39.5	4.75	0.98
	PMP 23/29/44		13.8	3.93	2.81	68.4	0.18	0.65	3.30	4.75	0.08	1.44	0.50	0.00	14.1	117	30.7	433	55.2	5.29	1.23
PMP-Ia Iberian	PMP 020		13.2	2.54	2.93	65.2	0.45	0.98	1.78	7.91	0.17	2.23	1.59	0.58	41.3	145	18.0	341	98.3	10.5	2.52
	PMP 15/16		17.2	2.55	3.51	63.8	0.63	1.16	2.01	7.06	0.25	0.04	1.63	0.00	34.0	135	26.5	279	141	12.8	2.92
PMP-Ib Iberian	PMP 045		18.4	4.22	1.78	61.8	0.42	1.24	1.71	7.63	0.08	1.21	0.72	0.35	47.3	185	8.75	425	20.7	4.63	1.18
	PMP 26/27/41/ 42	Cut decoration	15.7	2.28	1.36	71.8	0.32	1.13	1.75	4.05	0.07	0.61	0.66	0.19	35.7	110	6.32	265	21.6	4.07	1.00
PMP-S Sicily?	PMP 065		11.9	1.88	1.37	68.2	0.52	0.89	3.40	9.24	0.10	1.28	0.82	0.30	61.8	115	9.67	385	97.1	7.10	1.47
	PMP 048		15.5	2.28	1.31	64.9	0.42	0.64	2.62	10.0	0.10	1.22	0.73	0.04	61.8	97.3	8.30	415	98.8	7.60	1.47
	PMP 013		13.7	2.42	1.78	66.3	0.57	0.92	1.73	8.60	0.13	1.46	0.74	0.01	32.0	126	12.3	523	99.7	7.41	1.55

The high lead glass bead (PMP 001) differs from the soda-ash lead glass in that it has lower sodium (3%), phosphorus (0.2%) and magnesium (0.7%), and substantially higher aluminium (4%), potassium (1.6%), calcium (4%), titanium (0.15%) and iron oxides (5.3%). Elements related to the lead source such as arsenic (58 ppm), silver (6 ppm) and bismuth (4 ppm) are much lower than in most of the soda-ash lead samples, while it has notably higher barium (3383 ppm) (Additional file 1: Table S1). In these characteristics the bead resembles the high lead glass from Šaqunda (Cordoba) from the late eighth to early ninth century that is assumed to have been made from lead slag from silver or lead mining processes [3]. Despite the similarities in lead oxide contents with some North African Islamic beads [33], the black opaque bead from Cordoba has higher alkaline earth elements and significant differences in major and trace elements, particularly unusually high zinc contents.

### Plant ash glass

**Fig. 4:**
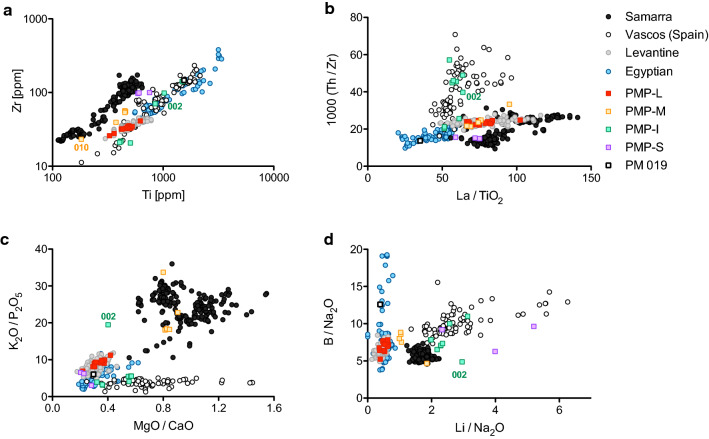
Different plant ash glass groups from Piscinas Municipales (PMP) compared to published glass reference groups. **a** Levantine plant ash glass is characterised by low zirconium and titanium contents, while sample PMP 019 has high Zr and Ti consistent with an Egyptian origin; **b** Th/Zr ratios distinguish plant ash glass produced in the Iberian Peninsula, highlighting once more the similarities between the bulk of the PMP samples with Levantine-type plant ash glass; **c** K_2_O/P_2_O_5_ versus MgO/CaO ratios clearly separate Mesopotamian and Iberian glasses from Levantine and Egyptian plant ash glass in terms of the plant ash component; **d** western Mediterranean and Mesopotamian glasses tend to have elevated Li relative to soda contents compared to glass from Egypt and the Levant, underscoring differences in the plant ash component and/or the production procedures. Data sources: Mesopotamian glass from Samarra [23], Egyptian plant ash glass weights [22], Tyre-type (P-1) plant ash glass from various Levantine sites [20, 34] and Iberian glass from Ciudad de Vascos [21]

The plant ash glass is characterised by high soda (Na_2_O > 10%) and elevated magnesia and potash contents (> 1.5%; Fig. 3). It is a diverse set of samples, indicating the use of different raw materials and production technologies. Alkali and alkaline earth metals (Li, B, Na, Mg, P, K, Ca) reflect the plant ash component. Together with elements associated with the silica source (Al_2_O_3_, TiO_2_, Zr, La, Th), the plant ash glass from Piscinas Municipales can be separated into at least four distinct compositional groups (Fig. 4; Table 1). By comparing these groups with published data of contemporary plant ash glasses from the Mediterranean and Mesopotamian region, we can then distinguish import from likely Iberian origin (Fig. 4). It is important to stress that any geographical attribution remains tentative and depends as much on the exclusion of potential sources as it does on positive attribution by way of compositional similarities.

The largest cluster of plant ash samples from Piscinas Municipales (PMP-L; n = 14) has characteristics that resemble those of early Islamic plant ash glass produced on the Levantine coast, specifically plant ash glass associated with the primary production site at Tyre that has been identified at various consumer sites throughout Syria-Palestine [16, 20, 22, 23]. This type of glass is generally low in silica related elements such as alumina (Al_2_O_3_ ≈ 1.75%), titanium (Ti < 700 ppm) and zirconium (Zr < 50 ppm; Fig. 4a), moderate alkali, alkaline earth elements and phosphorus, and typically low lithium and boron levels relative to soda (Fig. 4c–d). In contrast, three objects in yellow (PMP-M; PMP 023, 029 and 044 are from the same vessel) have much more pronounced plant ash characteristics such as high magnesium contents (MgO > 3.8%) and higher K_2_O/P_2_O_5_ ratios as well as higher lithium contents (Li > 10 ppm) consistent with plant ash glass from Mesopotamia represented by Samarra 1 and Samarra 2 that are believed to have been produced in the vicinity of Samarra (Iraq) (Fig. 4, Table 1) [18, 20, 23]. A single sample (PMP 019) with high zirconium (147 ppm) and titanium (1546 ppm) as well as higher boron to soda ratios matches Egyptian plant ash glass used for the production of tenth-century glass weights from Egypt (Fig. 4) [22]. Egyptian plant ash glass is similar to Levantine plant ash glass with respect to the alkali and alkaline earth signatures (Fig. 4c–d).

The remaining plant ash glasses do not correspond to any of the well-established compositional groups from the eastern Mediterranean (Levant, Egypt) or Mesopotamia. Four individual objects (eight analyses; PMP-I) have thorium to zirconium ratios and/or lithium to soda ratios that are notably higher than in any of the other plant ash groups and somewhat lower potash relative to phosphorus (Fig. 4b, d). Similar features have been observed in plant ash glass from Ciudad de Vascos, which was interpreted as an indication of an Iberian provenance of these glasses [21]. The Iberian soda-rich plant ash glasses from Piscinas Municipales are rather heterogeneous in terms of the elements related to the silica source. One sub-group (PMP-Ib) has low levels of aluminium, titanium and zirconium, whereas the other supposedly Iberian samples (PMP-Ia) have much higher concentrations of these elements (Table 1; Fig. 4b). This testifies to different raw materials and production events, and therefore different primary production locations. Sample PMP 002 has potassium values (K_2_O = 6.7%) that exceed those typically encountered in Mediterranean soda-ash glasses (Fig. 3), but it resembles other Iberian glasses with respect to the elevated thorium to zirconium ratios and lithium to soda ratios as well as other trace elements (Additional file 1: Table S1; Fig. 4b, d). The elevated potash levels may be the result of contamination by fuel ash during the manufacturing process [35].

Two colourless-greenish (PMP048 and PMP045) and one copper-blue sample (PMP013) do not fit into any of the eastern Mediterranean or Iberian groups (Table 1). Like the glasses with an Iberian signature, these three samples (PMP-S) have elevated lithium and higher phosphorus contents than the glass from the Levant, Egypt or Mesopotamia. They have higher zirconium, especially compared to titanium and thorium than most of the other regional production groups (Table 1, Fig. 4a–b). They resemble in these respects an Islamic glass assemblage from Mazara on the western coast of Sicily [24].

While the Levantine and Mesopotamian plant ash glasses are mostly colourless, with an occasional greenish tinge, the Iberian fragments are typically greenish in colour due to higher iron contents (Additional file 1: Table S1). Neither the Levantine nor the Mesopotamian glass exhibits obvious signs of recycling, except for PMP 035 that has minor lead contents (Additional file 1: Table S1). In contrast, the Iberian samples tend to have elevated lead and, in some cases copper, tin and/or antimony, probably due to the incorporation of some cullet during production.

In terms of the decorative techniques, the plant ash glasses are clearly distinct from the soda-ash lead glasses. All but one undecorated moulded unguentaria (PMP 012) are free-blown. The different plant ash groups are associated with different decorative techniques. Objects made of typical Iberian plant ash glass (PMP-Ia) have no decoration. The colourless fragments with wheel-cut decorations split into two compositional groups. PMP 014 and PMP 060 are plant ash glasses of Levantine origin and four fragments of the same vessel (PMP 026/027/041/042) were made from a relatively clean, presumably Iberian silica source (PMP-Ib; Additional file 2: Fig. S1). The same type of glass was used in a vessel of indeterminate shape, coloured in deep purple (PMP 045), a colour quite common in the glasses of al-Andalus [21]. The only flat glass fragment is of Levantine origin (PMP 024), and the only fragment with remains of gold leaf (PMP 010; Additional file 2: Fig. S1) comes from a luxury vessel of likely Mesopotamian provenance as it is compositionally very close to the glass from Samarra (Fig. 4a). Among the coloured glasses, the copper blue sample (PMP 013; Additional file 2: Fig. S1) has a composition similar to Sicilian glass.

## Discussion

### Production and distribution of soda-ash lead glass in al-Andalus

Our results show that the largest homogeneous compositional group among the domestic contexts from the tenth to early eleventh century in Cordoba was a very distinct soda-ash lead glass, consistent with other published results from Cordoba [3, 36]. This group was virtually unknown in the eastern Islamic world, but became widespread throughout the Iberian Peninsula between the tenth and twelfth centuries CE [21, 36]. It has been identified at various Islamic sites such as Ciudad de Vascos, Silves, Albalat [21], Toledo [37], Murcia [38, 39] as well as in the Christian contexts of Gauzon (Asturias) [40]. Nowhere else, however, does soda-ash lead glass attain the same prominence as in caliphal Cordoba, with the possible exception of Pechina (Almería) where about 37% of the analysed tenth-century samples are of a soda-ash lead composition [41].

A possible explanation for the dominance of soda-ash lead glass in both Cordoba and Almería could be the fact that both areas were major silver mining districts during the early Islamic period, comments about which can be found in contemporary Arab written sources [42, 43]. The recovery of metallic silver usually requires the cupellation of argentiferous lead ores, a process that results in a surplus of lead oxide in the form of litharge [31]. The trace element make-up of the soda-ash lead glass points to the use of litharge from silver processing, and lead isotope data have revealed the exploitation of various lead ore deposits in the southern Iberian Peninsula. While the lead component of the majority of soda-ash lead glass can be traced back to the region around Cordoba, the samples from Almería are isotopically closer to some mining districts in the south east of the peninsula, between Cartagena and Almería [30]. Furthermore, Cordoba and Pechina are the only two sites in al-Andalus where crucibles with leaded glass have thus far been found [41, 44].

**Fig. 5:**
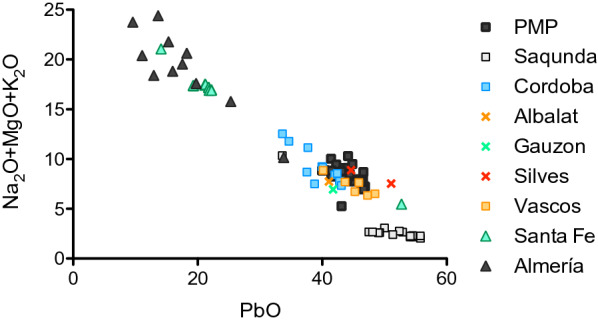
Sum of soda, potash and magnesia compared to lead contents of various soda-ash lead glasses. The soda-ash lead glass from Piscinas Municipales (dark grey squares) forms a neat cluster and compares well with soda-ash lead glasses from other sites in the Iberian Peninsula. Data sources: Albalat, Silves, Santa Fe (Schibille, unpublished), Saqunda [3], for Cordoba [36], for Vascos [21], for Gauzon [40], for Pechina/Almería [41], for Murcia [38, 39]

Figure 5 shows the combined soda, magnesia and potash versus the lead contents of the soda-ash lead glass from Piscinas Municipales (PMP) compared to soda-ash lead glasses recovered from other Iberian sites. The relative proportions of alkali and alkaline earths to lead contents imply differences in the glassmaking recipes. The glass from Almería [41] has the highest alkali and alkaline earths and lowest lead concentrations (the majority have PbO < 20%), while the lead slag glass from Saqunda [3] is at the other end with the highest lead (typically PbO > 50%) and only minor levels of alkali and alkaline earth metals. The glass from Piscinas Municipales and other sites in Cordoba [36] are close to the Saqunda glass with considerably higher lead (PbO > 40%) and lower alkali and alkaline earths than the glass from Almería (Fig. 5). Glass finds with similar lead levels have been identified in Ciudad de Vascos, Albalat, Gauzón, Santa Fe and Silves (Portugal). Soda-ash lead glasses with similar proportions of lead to alkali and alkaline earths as the glasses from Almería have so far only been found in Santa Fe (Toledo) and Murcia [38, 39]. These regional distribution patterns seem to indicate that soda-ash lead glass produced in or near Cordoba was traded more widely, to the northern and western regions of the Iberian Peninsula, whereas the soda-ash lead glass from Almería has a more limited range.

The ninth-century lead glass from Saqunda, as well as the slightly later soda-ash lead glass that dominates the archaeological record of Cordoba in the tenth century have no precedents in the glassmaking of the Islamic east or Carolingian Europe. Given similarities in composition and the use of the same lead source, it is reasonable to assume that soda-ash lead glass evolved from the earlier high lead slag glass found in Šaqunda, another suburb of Cordoba [3]. The emergence of a local high lead silica glass appears to be linked to shortages in the supply of natron glass from the eastern Mediterranean in the eighth and ninth centuries and the locally available glassy lead slag from silver and/or lead mining activities north of Cordoba. The earliest known soda-ash lead glass is a single sample from Šaqunda dated to the early ninth century [3], followed by several specimens in Pechina (Almería) in the second half of the ninth or early tenth century [41]. These developments were roughly contemporary to or possibly slightly earlier than the appearance of European types of high lead glass and potash lead glass in Carolingian Europe around the turn of the ninth century CE [4, 6, 45–47]. It is interesting to consider to what extent the developments of soda-ash lead glass in al-Andalus are related to these central European trends of wood-ash lead and so-called Slavic lead glass [4, 6]. Interactions between the different European glassmaking traditions is a tantalizing notion that deserves further investigation.

### Trade and local production of plant ash glass

The plant ash glasses from the Piscinas Municipales present a great internal variability indicative of various production centres (Egypt, Mesopotamia, Levant, Iberian Peninsula) in a more decentralised model than the one that had prevailed in late antiquity and earlier. The majority of the plant ash glasses seem to be of Levantine origin that may be a reflection of the relative significance of the glass industry in the Levant at the time. In the Near East, there is textual and archaeological evidence of long-distance trade of raw glass and cullet in the tenth and eleventh century [22, 48, 49]. No such large-scale imports of oriental glass are known from tenth-century al-Andalus, where imports pertained usually to finished products.

At the beginning of the eleventh century, Iberian plant ash glass still remains the exception in caliphal Cordoba. It appears that there was no plant ash glass production in Cordoba at the time. This is different to Almería where Iberian plant ash glass makes up almost half of the analysed assemblages and where hardly any imported soda-ash glass from other regions of the Islamic world has been identified for the same period [41]. Even though the earliest confirmed glass workshops in al-Andalus date to the twelfth century in Murcia [50, 51], the compositional evidence from Almería implies that primary production of plant ash glass had commenced by the tenth century and that it may have been located along the eastern coast of the Iberian Peninsula between Murcia/Alicante and Almería. What seems clear is that there were several active glassmaking centres in al-Andalus in the late tenth to early eleventh century, for both soda-ash lead as well as plant ash glass.

### Base glass types and glass decorating techniques

Mould-blown decorations account for about 50% of the decorated vessels in this study, and they are all made of soda-ash lead glass, confirming earlier suspicions that they were produced locally [10]. The earliest Iberian mould-blown decorated objects date from the tenth century and were used in al-Andalus throughout the Islamic period. Characteristic of the caliphal period (mid-10th/early 11th c.) are radially arranged drops or tears, mostly on drinking vessels (Fig. 2, PMP 007, 047, 057 and 062). Less common are radial ribs (PMP 017, 039) that seem to copy earlier Egyptian [52] or Levantine models [53]. The only other mould-blown vessels analysed from the Iberian Peninsula are those from Pechina (Almería) that were also made of soda-ash lead glass [41].

It appears, therefore, that the mould-blown technique was selectively used with this type of glass which may have technical, aesthetic and/or economic reason. Lead reduces the working temperature of glass and therefore the fuel costs. For example, glass containing 28% PbO has a working temperature range from 635 °C to 985 °C compared to an alkali lime silica glass that has a range from 715 °C to 1040 °C [54]. Lead also provides glass with a higher gloss which makes it suitable for decorated pieces. At the same time, lead decreases the viscosity, making it less suitable for free blowing. It was previously noted that Islamic high lead silica glass was used for specific types of decorated vessels such as cameo and a bright emerald green glass [5, 55]. In Egypt, in Rāya and Wadi al-Tūr, most of the lead silica glasses are cast with linear-cut and stamped decorations [32]. In Caesarea 50% of the high lead glasses were stamped, cut and/or mould decorated [55]. In both cases, it was suggested that the use of lead glass facilitated the deep-cut decorations due to the lower hardness of the material [5, 32, 56]. The only Islamic Iberian examples of embossed decorations analysed are the two fragments presented here (PMP 032 and 058), both made of soda-ash lead glass.

The low incidence of relief-cut and facet-cut vessels in al-Andalus and their stylistic and technical similarities with eastern models suggest that they were imported luxury goods from the Islamic East [8–13]. The relief-cut and linear cut glass vessels from the Iberian Peninsula so far analysed are all of eastern Mediterranean or Mesopotamian provenance. The fragments from Gauzón (Asturias), for example, were found to be of Levantine and Mesopotamian origin [40]. Of the three wheel-cut decorated objects recovered in Piscinas Municipales, two are Levantine imports (PMP 014, 060), while one bottle (PMP 026-27-41-42) probably represents a locally produced Iberian glass (Fig. 2, Table 1). It is a particularly clean glass with low levels of silica related impurities, but its thorium to zirconium ratios as well as the high lithium values nonetheless match those of typical Iberian glasses (Fig. 4). An Iberian origin is also consistent with the style of the relief-cut in that the outlines in the case of the bottle from Piscinas Municipales are not notched as is typically the case in relief-cut glass vessels from the eastern Mediterranean and Mesopotamia [14]. In short, neither the execution of the relief-cut nor the glass composition is compatible with a provenance in the eastern Mediterranean or Mesopotamia. The bottle from Piscinas Municipales thus provides the first concrete evidence that at least some relief-cut glass vessels were produced in Umayyad al-Andalus from locally sourced raw materials.

Finally, it is worth emphasising that glass objects decorated with gold leaf are extremely rare, both in the eastern Islamic world [52] as well as in Umayyad al-Andalus. The only known cases are a fragment from Madinat Ilbira from the ninth century [57], one from Cordoba from the tenth century [58], and the fragment found in Piscinas Municipales (PMP 010). The latter is the only one that has been analysed, and it has been found to be of Mesopotamian origin with typical high magnesium and low calcium, aluminium, phosphorus, titanium and zirconium contents [23].

## Conclusion

Glass finds from archaeological excavations are usually highly fragmented. The compositional evidence has allowed us to identify matching compositions of several fragments and thus to reconstruct the shape of some glass vessels, thereby extending the limited typological repertoire of glass from al-Andalus in the tenth century. This demonstrates a new, so far hardly explored possibility for the practical application of analytical methods to the study of glass assemblages.

The results have shown that local glassmaking was firmly established in al-Andalus by the tenth century. In Cordoba more than half of the glass finds were soda-ash lead glasses for which we have circumstantial and isotopic evidence that it was locally produced, exploiting lead sources from nearby mining districts north of the caliphal capital. The plant ash glass finds present an interesting mix of imported and locally produced objects. Given the heterogeneous nature of the plant ash glass, finished glass objects are likely to have arrived in al-Andalus from Egypt, the Levant, Mesopotamia and perhaps Sicily. Of particular importance was the confirmation that the characteristic mould-blown decorations, as suggested by some authors, where made locally from soda-ash lead glass. What is more surprising is that some of the wheel-cut luxury glass objects were probably also produced in al-Andalus. This is an issue that needs to be further explored in future research.

The analyses also revealed different types of Iberian plant-ash and soda-ash lead glass, which provides a clear indication of the co-existence of several glass production centres in al-Andalus and various distribution networks at that time. In the tenth and early eleventh century, plant ash glass of Mesopotamian and Mediterranean origin still played an important role in the supply of common goods and decorated luxury vessels alike. These imports have also been recorded in the north and central Iberian Peninsula. This testifies to regular trade with the east, which is mentioned in written sources, but has not yet been clearly confirmed on such a scale for any of the archaeological materials such as ceramics, metals or in fact glass. This illustrates once again the relevance of analytical studies for the evaluation of commercial long-distance networks and the extent of its markets.

## Supplementary Information

**Additional file 1: Table S1.** LA-ICP-MS data of Piscinas Municipales de Poniente glass, oxides and chlorine are given in [wt% ], elements in [ppm]. **Table S2.** Average LA-ICP-MS data of glass standards compared to published values for Corning reference glasses A, B, C, D, and NIST SRM 612.

**Additional file 2: Figure S1.** Selection of fragments representative of different base glass types and different decoration techniques.

## Data Availability

All relevant data and the references to the analysed materials are within the paper and its Additional files 1, 2.
