# Supplementary material for: Composition and origins of decorated glass from Umayyad Cordoba (Spain)
Source: Herit Sci. 2021 Mar 12;9(1):31. doi: 10.1186/s40494-021-00505-4 (PMC7954763; doi:10.1186/s40494-021-00505-4)
Supplement: Supplementary file 2 — Additional file 2: Figure S1. Selection of fragments representative of different base glass types and different decoration techniques. [file 40494_2021_505_MOESM2_ESM.pdf]

Supplementary material Figure S1: Selection of fragments representative of different base glass types and different decoration techniques.

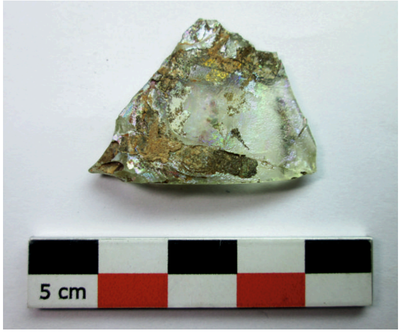

PMP 010 – Mesopotamian plant ash glass (PMP-M) with gold leaf decoration

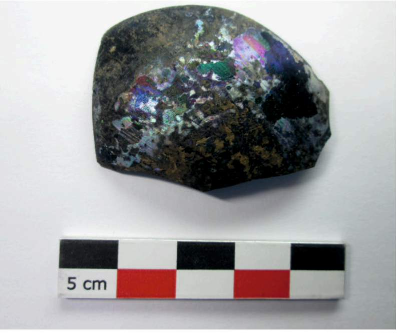

PMP 013 – dark blue Sicilian plant ash glass (PMP-S)

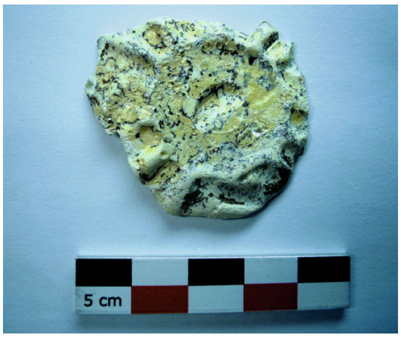

PMP 017 – soda-ash lead glass, mould blown

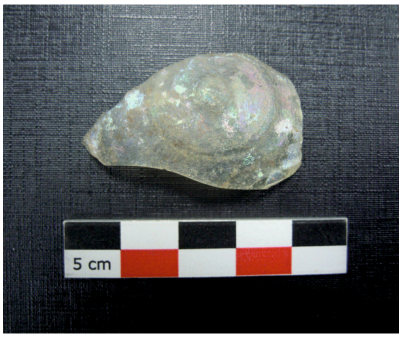

PMP 041 – Iberian plant ash glass (PMP-Ib) with cut decorations

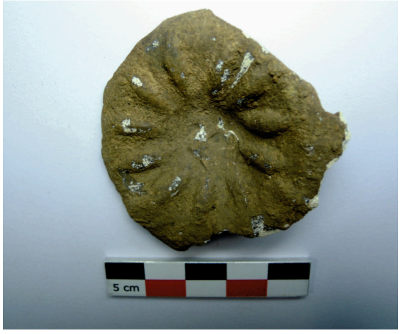

PMP 057 – soda-ash lead glass, mould blown

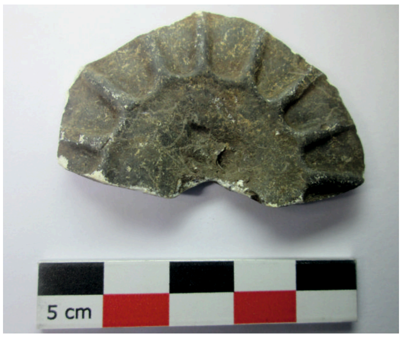

PMP 059 – soda-ash lead glass, mould blown

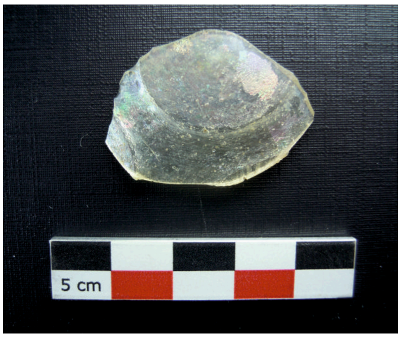

PMP 060 – Levantine plant ash glass (PMP-L) with cut decorations

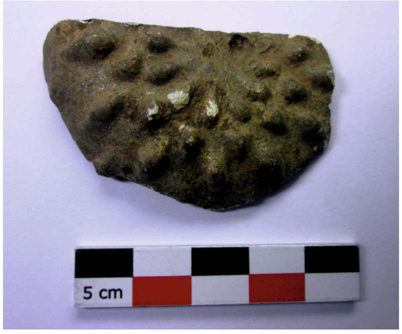

PMP 062 – soda-ash lead glass, mould blown
